# Diabetes Is Associated with Lower In-Hospital Mortality in Patients Undergoing Surgical Repair for Aortic Aneurysm Rupture

**DOI:** 10.3390/jcm14124370

**Published:** 2025-06-19

**Authors:** Hamza Chaudhry, Soha Dargham, Ziyad Mahfoud, Amin Jayyousi, Jassim Al Suwaidi, Charbel Abi Khalil

**Affiliations:** 1 Department of Genetic Medicine, Weill Cornell Medicine-Qatar, Doha P.O. Box 24144, Qatar; 2 Joan and Sanford I. Weill Department of Medicine, Weill Cornell Medicine, New York, NY 10065, USA; 3 Department of Medical Education, Weill Cornell Medicine-Qatar, Doha P.O. Box 24144, Qatar; 4 Biostatistics Core, Weill Cornell Medicine-Qatar, Doha P.O. Box 24144, Qatar; 5 Department of Endocrinology, Hamad Medical Corporation, Doha P.O. Box 3050, Qatar; 6 Heart Hospital, Hamad Medical Corporation, Doha P.O. Box 3050, Qatar

**Keywords:** aortic aneurysm, abdominal aortic aneurysm, thoracic aortic aneurysm, aortic aneurysm rupture, diabetes, cardiovascular disease

## Abstract

**Background**: Previous studies reported a protective effect of type 2 diabetes on the progression of aortic aneurysms. We aimed to investigate whether this paradoxical phenomenon remained in patients with diabetes undergoing repair of ruptured aortic aneurysms. **Methods**: Data from the US Nationwide Readmission Database from 2016 to 2019 were analyzed. Patients admitted for surgical repair of ruptured abdominal or thoracic aortic aneurysms were included. Patients discharged alive were followed for 30 days. The co-primary outcomes were in-hospital and 30-day mortality. **Results**: A total of 9858 patients hospitalized for surgical repair of ruptured abdominal or thoracic aortic aneurysm were included, of whom 16.4% had diabetes. A lower adjusted risk of in-hospital mortality in abdominal and thoracic aneurysms was observed in diabetes patients (aOR = 0.76 [0.67–0.87], 0.61 [0.46–0.810], respectively). However, atrial fibrillation and acute renal failure were more likely to occur in the presence of diabetes (aOR = 1.25 [1.11–1.42]; 1.17 [1.05–1.32], respectively). Within 30 days, diabetes was not associated with a difference in the incidence of mortality or readmission (aHR = 1.47 [95% CI 0.98–2.22]; 1.15 [95% CI 0.99–1.34], respectively). Cardiovascular system-related pathologies were the most prevalent etiologies in all readmitted patients. Infections were more likely to occur in the diabetes group (16.0% vs. 11.0%, respectively, *p* = 0.042). **Conclusions**: The paradoxical effect of diabetes is also observed in ruptured aneurysms treated surgically, as type 2 diabetes patients have a lower in-hospital mortality. However, this protective effect does not extend to 30-day readmission or survival.

## 1. Introduction

Aortic Aneurysm is a cardiovascular condition characterized by pathologic dilatation of an aortic segment due to weakening of the arterial wall, often defined as an aortic diameter greater than 50% of expected [1]. Aortic aneurysms are divided into three subtypes: abdominal aortic aneurysm (AAA), which is below the diaphragm, and thoracic aortic aneurysm (TAA), which is above the diaphragm; and thoracoabdominal aortic aneurysm (TAA). AAAs are 1.3–8.9% prevalent in men and 1.0–2.2% in women [2]. Smoking is the most substantial risk factor for AAAs, followed by age, with the incidence of AAAs significantly increasing after 55 years in men and after 70 years in women [3]. TAAs, on the other hand, are much less common; a recent meta-analysis determined the pooled incidence and prevalence to be 5.3 per 100,000 population [4]. TAAs are strongly associated with connective tissue disorders and non-syndromic familial patterns [3]. Other significant risk factors for both TAAs and AAAs include atherosclerosis, hypertension, and dyslipidemia [3].

Type 2 diabetes (T2D) is a significant risk factor for cardiovascular disease (CVD) and contributes to the atherosclerotic process [5]. Nevertheless, multiple epidemiological reports have found that T2D has an inverse association with aortic aneurysms [6]. Studies have shown that patients with diabetes develop smaller AAAs and lower growth rates of AAAs compared to those without diabetes [6].

Aneurysm rupture is a life-threatening emergency that requires immediate surgical intervention, with mortality rates reported between 40 and 80% [7]. However, there is a knowledge gap on the impact of diabetes on the post-repair of ruptured aneurysms. We aimed to compare in-hospital outcomes of patients with and without diabetes and analyze readmission trends post-aortic aneurysm rupture repair.

## 2. Patients and Methods

### 2.1. Data Source

Data were extracted from the US Nationwide Readmissions Database (NRD) from 1 January 2016 to 31 December 2019. The NRD was developed by the Healthcare Cost and Utilization Project (HCUP) and is sponsored by the Agency for Healthcare Research and Quality (AHRQ) [8]. The database contains data on all-payer hospital inpatient stays from hospitals in 31 states, accounting for 62.2 percent of the total U.S. resident population and 60.8 percent of all U.S. hospitalizations [9]. The NRD provides comprehensive data on patient demographics, discharge outcomes, and readmission data. Since 2016, all diagnoses and procedures have been encoded using the International Classification of Diseases, Tenth Revision (ICD-10) [9].

### 2.2. Study Population and Outcomes

We included patients with a primary diagnosis of ruptured abdominal (ICD-10 code: I71.30, I71.31, I71.32, and I71.33) or thoracic aneurysm (ICD-10 code: I71.10, I71.11, I71.12, and I71.13) undergoing a repair between 2016 and 2019 and then stratified based on the presence of T2D. These patients comprised our index group. Patients who survived the repair and were discharged alive from the hospital were followed for 30 days, constituting our readmission group. We excluded patients with a ruptured thoracoabdominal aneurysm or at an unspecified site. Those under 18 or with missing data regarding their age, mortality, or gender were also excluded. The primary outcome for the index group was in-hospital mortality, and the co-primary outcome for the readmitted group was 30-day mortality. Secondary outcomes were in-hospital atrial fibrillation (AF), acute renal failure (ARF), and 30-day readmission. In-hospital outcomes occurred during or after surgery. 30-day mortality and readmission occurred within 30 days post-discharge.

### 2.3. Statistical Analysis

We first compared the baseline characteristics and in-hospital outcomes of patients with ruptured aneurysms with and without diabetes, followed by an assessment of the predictors of these outcomes. Next, we analyzed the baseline characteristics and 30-day outcomes of patients discharged alive after the repair of ruptured aneurysms, stratified by diabetes status, and assessed the predictors of these outcomes. Finally, we examined the etiologies of 30-day readmission. Patients’ demographics and comorbidities were presented as numbers and percentages. Statistical comparisons between diabetes and non-diabetes patients were conducted using the Pearson chi-square or Student *t*-test. Odds ratios and their corresponding confidence intervals were computed using binary logistic regression. Further adjustments were made in age, sex, and baseline characteristics between groups, which were obesity, hypertension, smoking, dyslipidemia, chronic kidney disease (CKD), and coronary artery disease (CAD). Kaplan-Meier curves were also reported for mortality and readmission, stratified by diabetes. Hazard ratios were calculated for mortality and readmission using the log-rank tests and then corrected for parameters that differed between groups using a Cox regression hazard model. A *p*-value of less than 0.05 was considered statistically significant. Statistical analysis was conducted through SPSS Statistics version 26.0 (IBM, Armonk, NY, USA, version 26.0).

## 3. Results

### 3.1. Study Population

**Figure 1:**
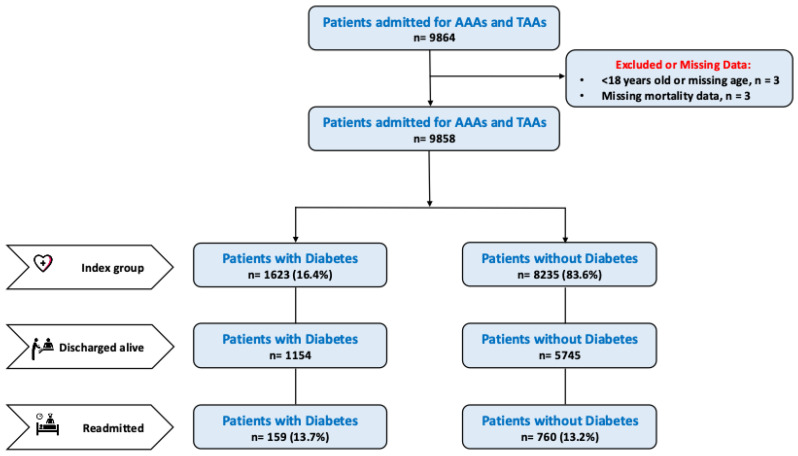
Flow chart of the study.

A total of 9858 cases (index group) were extracted, of whom 8235 patients (83.6%) didn’t have diabetes and 1623 (16.4%) had it. Within 30 days, 219 patients (13.4%) from the diabetes group and 760 (9.2%) from the non-diabetes group were readmitted (readmission group) (Figure 1). The mean age of patients with diabetes was not significantly different from that of patients without diabetes (74 (9) vs. 73 (11), respectively, *p* = 0.179) (Table 1). Notably, non-diabetes had a larger percentage of patients at the extremes of the age distribution (age <55 and age >84) than the diabetes group. There was a more significant proportion of males in the diabetes population, with 72.1% compared to 69.4% in the non-diabetes population (*p* = 0.036). Patients with diabetes had a longer length of stay (6 [2,3,4,5,6,7,8,9,10,11,12] days), in contrast to patients without diabetes (5 [1,2,3,4,5,6,7,8,9,10] days) (*p* < 0.001). As expected, a higher percentage of comorbidities is observed in the diabetes group.

### 3.2. In-Hospital Outcome

469 (28.9%) of diabetes patients and 2940 (35.7%) of non-diabetes patients died; hence, diabetes was associated with a lower adjusted risk of mortality (aOR = 0.85 [0.75–0.96]) (Table 2). However, atrial fibrillation and acute renal failure were more likely to occur in the presence of diabetes (aOR = 1.25 [1.11–1.42]; 1.17 [1.05–1.32], respectively). Further stratified analysis showed that the lower mortality risk was present in TAAs (aOR = 0.61 [0.46–0.810]) and AAAs (aOR = 0.76 [0.67–0.87]). The protective effect of diabetes was also not modified according to gender (*p* interaction = 0.52), age (*p* interaction = 0.43), or the presence of obesity (*p* interaction = 0.11), hypertension (*p* interaction 0.63), or dyslipidemia (*p* interaction = 0.55). As expected, diabetes patients who smoke have a higher mortality risk (OR = 2.27 [95% CI 1.13–4.53]).

Both groups’ predictors of mortality, AF and ARF, are shown in Supplementary Table S1. Patients above the age of 84 had an increased risk for mortality of almost 4-fold in both groups. Females, with and without diabetes, were more prone to die (OR = 1.51 [1.19–1.90]; OR = 1.68 [1.52–1.85], respectively). Obesity was found to decrease the risk of mortality in both populations (OR = 0.75 [0.56–0.99] and 0.69 [0.59–0.80], diabetes and non-diabetes, respectively). Interestingly, obesity, not hypertension or dyslipidemia, protected all patients. Patients above 65 years of age were four times more likely to develop AF in the diabetes group and two times in the non-diabetes group. CKD and VHD also increased the risk of AF in all patients. Females were less likely to develop ARF in the diabetes and non-diabetes populations (OR = 0.77 [0.62–0.96]; OR = 0.62 [0.56–0.68], respectively). Obesity increased the risk of ARF in the non-diabetes population only (OR = 1.57 [1.37–1.81]). Unsurprisingly, CKD significantly increased the likelihood of developing ARF in both populations (OR = 2.76 [2.23–3.41]; OR = 3.09 [2.77–3.44], diabetes and non-diabetes, respectively).

### 3.3. Readmission

**Figure 2:**
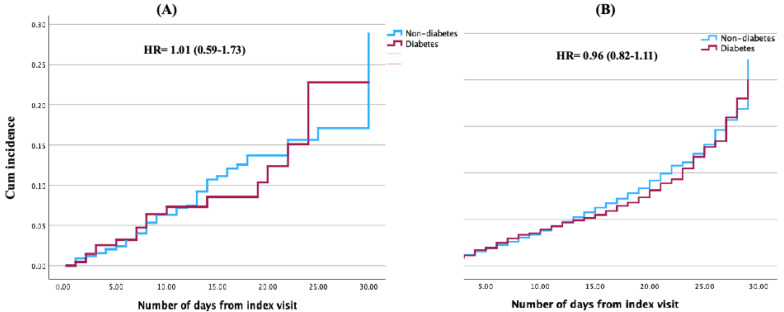
Cumulative incidence curve of (**A**) mortality and (**B**) readmission.

Patients readmitted within 30 days of discharge were analyzed. One hundred fifty-nine patients from the diabetes group (13.7%) and 760 from the control group (13.2%) were readmitted within 30 days of discharge. Demographics, comorbidities, and outcomes were re-compared between these subgroups (Supplementary Table S2). There was a significantly higher percentage of younger patients (age <55) in the non-diabetes group (6.7% compared to 2.3%, *p* < 0.001). Diabetes patients had a higher prevalence of obesity (19.6% to 8.7%), hypertension (91.3% to 82.2%), and chronic kidney disease (38.8% to 27.8%) (*p* < 0.001 for all). There was no significant difference in the incidence of 30-day mortality or 30-day readmission rate between both groups (Figure 2), even after adjustment (cox proportional HR = 1.15 [0.65–2.02]; HR = 0.95 [0.82–1.11], mortality and readmission, respectively).

We evaluated the etiologies of 30-day readmission. Cardiovascular system-related pathologies were the most prevalent in both groups, with 26% in diabetes (Supplementary Figure S1A) and 30% in non-diabetes (Supplementary Figure S1B) (*p* = 0.32). Infections were more prevalent in the diabetes group than in the non-diabetes group (16.0% vs. 11%, respectively, *p* = 0.042). We further looked at the most common cardiovascular-related etiologies. Hypertensive heart disease with heart failure was more prevalent in patients with diabetes (12%) (Supplementary Figure S1C) than in those without (6%) (*p* = 0.043). (Supplementary Figure S1D). Twelve non-diabetic (5%) and four diabetic patients (7%) were readmitted because of a secondary intervention (*p* = 0.15).

## 4. Discussion

In this large database, we report that diabetes is associated with lower mortality in patients operated for a ruptured aortic aneurysm. To the best of our knowledge, we are the first to report that this protective impact of diabetes fades within 30 days of the operation. In line with the interest in the impact of diabetes on aortic disease, our recent findings indicate that diabetes does not increase in-hospital or short-term mortality among patients undergoing surgical repair for type A aortic dissection [10].

Although data on the cardiovascular outcomes of surgical repair of aneurysm rupture in diabetes patients is scarce, several studies and meta-analyses on the topic concluded that patients with diabetes had a significantly lower mortality related to the prevalence and incidence of aneurysms [11]. A single-center study found that aortic aneurysm rupture-related mortality was lower in patients with diabetes [12]. However, the study above included only thirteen diabetes patients with a ruptured aneurysm. In a retrospective French multicenter study, a higher in-hospital mortality rate was reported in diabetes patients who underwent open or endovascular repair for unruptured aortic aneurysms [13]. A similar Swedish study did not find an impact of diabetes on the rates of acute myocardial infarction and stroke following the repair of ruptured aneurysms [14]. Our study found that female patients had higher mortality post-aortic aneurysm rupture than males, which is consistent with the literature that indicates females have higher mortality and post-operative mortality for ruptured aneurysms [15]. Among readmitted patients, the ones with diabetes were more likely to have post-operative infections, which is concordant with previous results reporting a high predisposition to infections and delayed wound healing postoperatively in the presence of diabetes [16,17], which may offset the initial in-hospital protective effect observed in this population.

It is not clearly understood why diabetes reduces the incidence of aneurysm rupture and is associated with lower mortality in case of a rupture. One study observed a negative correlation between fasting glucose levels and aortic diameter [18]. A sub-analysis of the VIVA trial, a randomized controlled screening trial for aortic aneurysms in Denmark, demonstrated that elevated HbA1c is linked to decreased aneurysmal growth [19]. Certain diabetes medications have been associated with a potentially protective effect against the development and progression of aortic aneurysms [20]. Metformin has been observed in some studies to reduce the growth rate of abdominal aortic aneurysms, possibly due to its effects on reducing inflammation and oxidative stress in the vascular system [21]. Experimental studies assessing SGLT2 inhibitors and GLP-1 agonists have suggested a protective effect, although epidemiological data to confirm these findings are lacking [22,23]. Some studies have also indicated that in experimental settings, thiazolidinediones may protect against aortic aneurysm development or progression by reducing inflammation within the aortic wall [24]. The favorable impact of other diabetes medications, such as insulin, sulfonylureas, and DPP-IV inhibitors, on aneurysm progression remains unclear due to the limited number of small-scale studies. One plausible explanation for the protective impact of diabetes lies within the vascular wall, as the extracellular matrix has a high content of fibrous proteins, thereby increasing the intima-wall thickness and protecting it from rupture [6]. Hyperglycemia leads to the formation of advanced glycation end-products, which cause collagen cross-linking [25]. This cross-linking increases the stiffness of the aortic wall, making it more resistant to the dilation seen in aneurysms [26]. However, other studies suggest that vessel calcification has a superior prognostic value than diabetes in predicting cardiovascular outcomes [27].

We acknowledge the presence of limitations in our study. Although the NRD is a large cohort, a disadvantage is that the data is collected for reimbursement purposes rather than clinical practice. Additionally, there is a possibility of incomplete data within the database, which may lead to inaccurate results. In this analysis, we examined only patients who reached the operating room alive, and there may be differences in the proportion of patients with and without diabetes who succumb before hospital admission. Neither do we know if the surgeons employ the same criteria when selecting which patients with ruptured TAAs or AAAs, with and without diabetes, are considered fit for surgery. It also remains unknown whether aneurysms were repaired by surgery or endovascular treatment, as the type of repair is present in fewer than 50% of patients. In a recent meta-analysis of randomized controlled trials comparing open surgery to endovascular treatment in nearly 3000 patients, the endovascular approach was associated with a better outcome during the first six months but carried an increased mortality risk after eight years [28]. The NRD data elements used did not include race, which may play a role in accounting for differences between the two groups. It has recently been shown that African Americans were less likely to have ultrasound scans during follow-ups of aneurysms [29]. Cardioprotective medications, such as beta-blockers and angiotensin II receptor blockers (ARBs), are also missing on admission in all patients. Those medications are commonly prescribed to lower blood pressure and reduce the stress on the aortic wall, thereby slowing the aneurysm’s growth [30]. Furthermore, several studies have shown that beta-blockers are associated with a significant reduction in postoperative mortality [30,31]. Importantly, for patients with diabetes, the NRD does not report key diabetes characteristics, such as glycemic control, diabetes duration, or medication use. Finally, factors that affect short-term outcomes, such as preoperative cardiac arrest, body temperature, base deficit, and hemodynamic instability, were also not included in the database. Nevertheless, we believe that our study has several strengths, including a large sample size that is representative of the US population. It also explored an important paradox in vascular surgery outcomes.

## 5. Conclusions

In this analysis of patients with aortic aneurysm rupture treated surgically, we observed a lower mortality risk in patients with diabetes. However, diabetes did not significantly impact 30-day mortality or readmission. As the global population ages and the prevalence of diabetes continues to rise, understanding the relationship between diabetes and aneurysms is valuable. Further studies that can factor in medical treatment, specific diabetes parameters, and racial background could offer a better understanding of the proper relationship between diabetes and aortic aneurysms.

## Figures and Tables

**Table 1:** Comparison of baseline characteristics of patients with ruptured aneurysm with vs. without diabetes.

	Diabetes n = 1623	Non-Diabetes n = 8235	*p*-Value
**Age**			
<55	38 (2.3%)	371 (4.5%)	**<0.001**
55–64	248 (15.3%)	1291 (15.7%)	
65–74	517 (31.9%)	2493 (30.3%)	
75–84	562 (34.6%)	2396 (29.1%)	
>84	258 (15.9%)	1684 (20.4%)	
Mean (SD)	74.2 (9.8)	73.8 (11.2)	**0.179**
**Sex**			
Male	1168 (72.1%)	5711 (69.4%)	**0.036**
**Income**			
Low	430 (26.8%)	2062 (25.4%)	**0.527**
Low-Mid	464 (28.9%)	2295 (28.3%)	
High-Mid	401 (25.0%)	2127 (26.2%)	
High	311 (19.4%)	1624 (20.0%)	
**Comorbidities**			
Obesity	312 (19.2%)	910 (11.1%)	**<0.001**
Hypertension	1437 (88.5%)	6109 (74.2%)	**<0.001**
Smoking	502 (30.9%)	2036 (24.7%)	**<0.001**
Dyslipidemia	892 (55.0%)	3084 (37.4%)	**<0.001**
VHD	120 (7.4%)	629 (7.6%)	**0.734**
CKD	535 (33.0%)	1723 (20.9%)	**<0.001**
CAD	9 (0.6%)	16 (0.2%)	**0.008**
**Location of Aneurysm**			
Abdominal Aorta	1331 (82.0%)	6770 (83.2%)	**0.849**
Thoracic Aorta	292 (18.0%)	1465 (17.8%)	
**Length of Stay** (days)	6 (2–12)	5 (1–10)	**<0.001**

CAD coronary disease, CKD chronic kidney disease, VHD valvular heart disease.

**Table 2:** Comparison of in-hospital outcomes in patients with ruptured aneurysms with and without diabetes.

	Number of Events				
Outcomes	Diabetes	Non-Diabetes	Unadjusted OR (95% CI)	Adjusted OR (95% CI)	*p*-Value
Mortality	469 (28.9%)	2940 (35.7%)	0.73 (0.65–0.82)	0.85 (0.75–0.96)	**<0.001**
Atrial Fibrillation	483 (29.8%)	1887 (22.9%)	1.43 (1.26–1.60)	1.25 (1.11–1.42)	**<0.001**
Acute Renal Failure	725 (44.7%)	3301 (40.1%)	1.21 (1.08–1.34)	1.17 (1.05–1.32)	**<0.001**

## Data Availability

The original contributions presented in this study are included in the article. Further inquiries can be directed to the corresponding author.

## Supplementary Materials

The following supporting information can be downloaded at: https://www.mdpi.com/article/10.3390/jcm14124370/s1, Figure S1: Etiology of readmission in patients (A) with diabetes and (B) without diabetes. Cardiovascular etiology of readmission in patients (C) with diabetes and (D) without diabetes; Table S1: Predictors of in-hospital outcomes compared by the presence of diabetes; Table S2: Baseline characteristics of patients readmitted within 30 days, stratified by the presence of diabetes.

## Abbreviations

AAA	Abdominal Aortic Aneurysm
AF	Atrial fibrillation
ARF	Acute renal failure
TAO	Thoracic Aortic Aneurysm
T2D	Type 2 diabetes
CVD	Cardiovascular disease
NRD	National Readmission Database
