# Supplementary material for: Diabetes Is Associated with Lower In-Hospital Mortality in Patients Undergoing Surgical Repair for Aortic Aneurysm Rupture
Source: J Clin Med. 2025 Jun 19;14(12):4370. doi: 10.3390/jcm14124370 (PMC12194128; doi:10.3390/jcm14124370)

Supplementary Table S1: Predictors of in-hospital outcomes compared by the presence of diabetes.

		Mortality				Atrial Fibrillation				Acute Renal Failure			
		Diabetes		Non-diabetes		Diabetes		Non-diabetes		Diabetes		Non-diabetes	
Predictors		**OR (95% CI)**	**P-value**	**OR (95% CI)**	**P-value**	**OR (95% CI)**	**P-value**	**OR (95% CI)**	**P-value**	**OR (95% CI)**	**P-value**	**OR (95% CI)**	**P-value**
Age	**<55 (ref)**												
	55-64	1.12 (0.44-2.84)	0.813	1.17 (0.88-1.56)	0.279	1.98 (0.58-6.78)	0.276	0.99 (0.68-1.44)	0.960	1.93 (0.88-4.26)	0.102	0.99 (0.78-1.25)	0.907
	65-74	1.77 (0.73-4.34)	0.209	1.62 (1.23-2.12)	<0.001	4.46 (1.35-14.73)	0.014	2.07 (1.47-2.91)	<0.001	2.84 (1.32-6.11)	0.008	1.10 (0.88-1.37)	0.412
	75-84	2.53 (1.04-6.17)	0.041	2.82 (2.15-3.68)	<0.001	6.64 (2.01-21.89)	0.002	3.21 (2.28-4.50)	<0.001	3.22 (1.50-6.93)	0.003	0.97 (0.78-1.21)	0.772
	>84	3.96 (1.60-9.81)	0.003	4.62 (3.52-6.07)	<0.001	7.03 (2.11-23.47)	0.002	3.93 (2.79-5.54)	<0.001	2.04 (0.93-4.49)	0.077	0.84 (0.71-1.11)	0.310
Sex	**Male (ref)**												
	Female	1.51 (1.19-1.90)	<0.001	1.68 (1.52-1.85)	<0.001	1.09 (0.86-1.37)	0.499	1.02 (0.91-1.14)	0.706	0.77 (0.62-0.96)	0.018	0.62 (0.56-0.68)	<0.001
Income	**Low (ref)**												
	Low-Mid	0.96 (0.71-1.27)	0.728	1.19 (1.05-1.35)	0.006	1.29 (0.97-1.73)	0.084	1.02 (0.88-1.18)	0.839	0.87 (0.66-1.13)	0.284	1.10 (0.97-1.24)	0.139
	High-Mid	1.01 (0.75-1.36)	0.973	1.09 (0.96-1.24)	0.195	1.16 (0.85-1.57)	0.354	1.30 (1.13-1.51)	<0.001	1.14 (0.87-1.50)	0.342	1.03 (0.91-1.17)	0.608
	High	1.00 (0.73-1.39)	0.955	1.23 (1.08-1.41)	0.003	1.51 (1.10-2.07)	0.012	1.34 (1.15-1.56)	<0.001	0.86 (0.64-1.15)	0.306	1.01 (0.88-1.15)	0.914
Obesity	**No (ref)**												
	Yes	0.75 (0.56-0.99)	0.049	0.69 (0.59-0.80)	<0.001	1.16 (0.89-1.52)	0.262	1.14 (0.97-1.34)	0.103	1.26 (0.99-1.62)	0.064	1.57 (1.37-1.81)	<0.001
Hypertension	**No (ref)**												
	Yes	0.96 (0.83-1.21)	0.654	0.97 (0.81-1.33)	0.734	1.33 (0.94-1.89)	0.112	1.77 (1.55-2.01)	<0.001	0.87 (0.64-1.18)	0.354	0.93 (0.84-1.03)	0.153
Smoking	**No (ref)**												
	Yes	0.83 (0.66-1.05)	0.122	0.91 (0.81-1.01)	0.053	1.05 (0.84-1.32)	0.672	1.42 (1.26-1.59)	<0.001	0.88 (0.73-1.06)	0.224	0.95 (0.89-1.09)	0.407
Dyslipidemia	**No (ref)**												
	Yes	0.83 (0.67-1.03)	0.083	0.68 (0.62-0.75)	<0.001	1.08 (0.87-1.34)	0.475	1.57 (1.42-1.74)	<0.001	0.89 (0.75-1.05)	0.179	0.92 (0.82-1.01)	0.075
VHD	**No (re**f)												
	Yes	0.70 (0.45-1.09)	0.110	0.69 (0.58-0.83)	<0.001	1.98 (1.35-2.88)	<0.001	3.34 (2.83-3.94)	<0.001	0.62 (0.42-0.92)	0.017	0.95 (0.80-1.12)	0.546
CKD	**No (ref)**												
	Yes	1.00 (0.80-1.26)	0.963	0.99 (0.89-1.11)	0.859	1.55 (1.24-1.94)	<0.001	1.80 (1.60-2.02)	<0.001	2.76 (2.23-3.41)	<0.001	3.09 (2.77-3.44)	<0.001
CAD	**No (ref)**												
	Yes	0.70 (0.15-3.39)	0.659	1.80 (0.68-4.81)	0.239	1.90 (0.51-7.09)	0.342	3.37 (1.27-9.00)	0.015	0.99 (0.27-3.70)	0.989	0.90 (0.33-2.47)	0.833

CAD= coronary disease, CKD= chronic kidney disease, VHD= valvular heart disease

Supplementary Table S2: Baseline characteristics of patients readmitted within 30 days, stratified by the presence of diabetes.

Year	Diabetes<br>n =219	Non-Diabetes<br>n = 760	P-value
**Age**			
<55	5<br>(2.3%)	51<br>(6.7%)	**0.062**
55-64	37<br>(16.9%)	133<br>(17.5%)	
65 - 74	78<br>(35.6%)	251<br>(33.0%)	
75-84	75<br>(34.2%)	219<br>(28.8%)	
>84	24<br>(11.0%)	106<br>(13.9%)	
Mean (SD)	73.10 (9.5)	71.96 (11.2)	**0.171**
**Sex**			
Male	159<br>(72.6%)	525<br>(69.1%)	**0.317**
**Income**			
Low	65<br>(30.0%)	186<br>(24.9%)	**0.300**
Low-Mid	58<br>(26.7%)	201<br>(26.9%)	
High-Mid	48<br>(22.1%)	207<br>(27.7%)	
High	46<br>(21.2%)	154<br>(20.6%)	
**Comorbidities**			
Obesity	43<br>(19.6%)	66<br>(8.7%)	**<0.001**
Hypertension	299<br>(91.3%)	625<br>(82.2%)	**<0.001**
Smoking	76<br>(34.7%)	268<br>(35.3%)	**0.878**
Dyslipidemia	113<br>(51.6%)	345<br>(45.4%)	**0.105**
VHD	23<br>(10.5%)	84<br>(11.1%)	**0.818**
CKD	85<br>(38.8%)	211<br>(27.8%)	**<0.001**
CAD	0<br>(0%)	2<br>(0.3%)	**0.447**
**Location of Aneurysm**			
Abdominal Aorta	175<br>(79.9%)	595<br>(78.4%)	**0.629**
Thoracic Aorta	44<br>(20.1%)	164<br>(21.6%)	
**Length of Stay** (days)	5 (3-11)	4 (3-9)	**0.113**

CAD= coronary disease, CKD= chronic kidney disease, VHD= valvular heart disease

Figure S1: Etiology of readmission in patients (**A**) with diabetes and (**B**) without diabetes. Cardiovascular etiology of readmission in patients (**C**) with diabetes and (**D**) without diabetes.

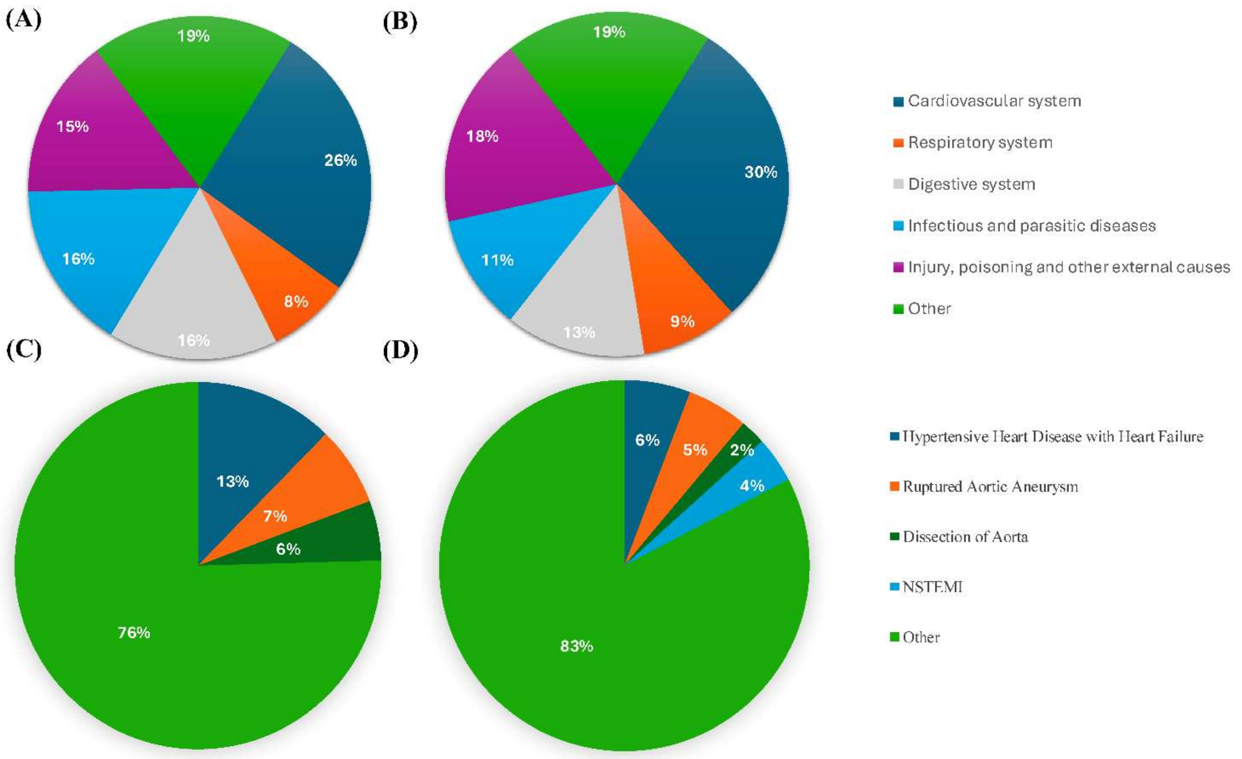

Supplement: Supplementary file 1 [file jcm-14-04370-s001.zip › jcm-3685510-supplementary.pdf]
